# Linalool Odor-Induced Anxiolytic Effects in Mice

**DOI:** 10.3389/fnbeh.2018.00241

**Published:** 2018-10-23

**Authors:** Hiroki Harada, Hideki Kashiwadani, Yuichi Kanmura, Tomoyuki Kuwaki

**Affiliations:** ^1^Department of Physiology, Graduate School of Medical and Dental Sciences, Kagoshima University, Kagoshima, Japan; ^2^Department of Anesthesiology, Graduate School of Medical and Dental Sciences, Kagoshima University, Kagoshima, Japan

**Keywords:** linalool, odor, anxiety, anxiolytic, benzodiazepine, flumazenil

## Abstract

In folk medicine, it has long been believed that odorous compounds derived from plant extracts can have anxiolytic effects. Among them, linalool, one of the terpene alcohols in lavender extracts, has been reported to have the anxiolytic effects. However, the anxiolytic nature of the linalool odor itself as well as its potential action through the olfactory system has not been thoroughly examined. In this study, we examined the anxiolytic effects of linalool odor with light/dark box test and with elevated plus maze (EPM), and found that linalool odor has an anxiolytic effect without motor impairment in mice. The effect was not observed in anosmic mice, indicating that it was triggered by olfactory input evoked by linalool odor. Furthermore, the effect was antagonized by flumazenil, indicating that the linalool odor-induced anxiolytic effect was mediated by γ-aminobutyric acid (GABA)ergic transmission via benzodiazepine (BDZ)-responsive GABA_A_ receptors. These results provide information about the potential central neuronal mechanisms underlying the odor-induced anxiolytic effects and the foundation for exploring clinical application of linalool odor in anxiety treatments.

## Introduction

Anxiety disorders are the most prevalent class of mental disorders. About 5.3% of adults in Japan or 18.2% of adults in the USA meet the diagnostic criteria for at least one anxiety disorder within the past 12-months (Demyttenaere et al., 2004). Due to these high rates, the development of effective therapy and therapeutic tools for treating anxiety disorders is the one of the more pressing issues in the field of mental science.

Therapy utilizing anxiolytic drugs has long been a first-line choice as an effective treatment options for anxiety disorders (Hoffman and Mathew, 2008). The most well-developed and commonly prescribed drugs used to treat anxiety disorders are azapirons and serotonin selective reuptake inhibitors (SSRIs), which modulate serotonergic synaptic transmission, and benzodiazepines (BDZs), which modulates γ-aminobutyric acid (GABA)ergic (Ravindran and Stein, 2010). However, the side effects (e.g., clinical effect delay, headache, somnolence, dizziness and serotonin syndrome for azapirones; clinical effect delay, sexual dysfunction and serotonin syndrome for SSRI; abuse, dependence liability, retrograde amnesia, and sedation for BDZs) of these drugs can be severe and more detrimental than the anxiety itself so further development of new drugs is still expected and necessary (Nash and Nutt, 2005).

In addition to anxiolytic pharmaceuticals, aromatic compounds derived from plant extracts have been used in traditional medicine as a treatment for anxiety (Connor and Vaishnavi, 2009). For example, lavender extract has been used to treat patients suffering from anxiety (Kasper et al., 2010). Several compounds extracted from lavender, such as linalool, were reported to have anxiolytic effects (De Sousa et al., 2015). However, the neuronal mechanisms underlying the reported anxiolytic effects of the odorous compounds have not yet been fully revealed. In the case of linalool, the anxiolytic effects of the odor itself have also not yet been addressed.

In this study, we examined the anxiolytic effects of linalool odor in mice. Classical anxiety-related behavioral tests showed that exposure to linalool odor induced significant anxiolytic effects. The effects were not observed in anosmic mice, indicating that the effects were triggered by olfactory input evoked by linalool odor. Furthermore, we found that flumazenil antagonized the linalool odor-induced anxiolytic effects, indicating that BDZ-sensitive GABAergic transmission plays a pivotal role for the anxiolytic effects.

## Materials and Methods

### Animals

Male wild type mice (C57BL/6N, 25–35 g, *n* = 240) originally purchased from CLEA Japan (Tokyo, Japan) were used to avoid possible variations related to menstrual cycling in females. All animals were maintained under a constant temperature (24 ± 1°C) with free access to food and water. Animals were housed with lights on at 7:00 A.M. and off at 7:00 P.M. All experiments were performed during the light cycle, between 12:00 P.M. and 5:00 P.M. Animals were naive to linalool odor and drugs, and each mouse was used only once to avoid carry-over effects. Animals were acclimatized over 3 days with 3 min of handling on each day. On experiment days, mice were moved to the experiment room 3 h prior to the start of the experiment. All experiments were performed in accordance with guidelines outlined by the Physiological Society of Japan and were approved by the Experimental Animal Research Committee of Kagoshima University.

### Drugs

Cercine^®^ (5 mg/mL Diazepam (positive allosteric modulator for γ-aminobutyric acid A receptors (GABA_A_Rs) with BDZ binding site, 1.5 mg/kg i.p.; Takeda Pharmaceutical Co., Ltd., Osaka, Japan)), Flumazenil (selective antagonist for BDZ site of GABA_A_Rs 3 mg/kg i.p.) and WAY100635 (antagonist for serotonin 1A receptor (5-HT_1A_R), 0.5 mg/kg i.p.; Tocris Bioscience, Boston, MO, USA) were purchased. Diazepam was diluted with 0.9% NaCl. Flumazenil and WAY100635 were dissolved in Tween80 and diluted with 0.9% NaCl (final concentration of Tween80 was 2%). All drugs and vehicle (2% Tween80 in 0.9% NaCl) were injected intraperitoneally 30 min before behavioral tests. Linalool was purchased from Tokyo Chemical Industry. 3-methylindole (3-MI; 300 mg/kg i.p.; Sigma, St. Louis, MO, USA) was injected intraperitoneally for olfactory epithelium deprivation. Corn oil was used as vehicle for 3-MI.

### Linalool Odor Exposure

Linalool odor exposure was performed in a custom-made odor chamber. A piece of 2 cm × 2 cm filter paper treated with 0, 20, 200, or 2,000 μL of linalool was placed at each of the four corners of an acryl box (25 cm × 25 cm × 25 cm). A mouse was placed into an acryl cage with a wire netting cover (12 cm × 20 cm × 10 cm) and was placed at the center of the odor chamber. Mice were unable to access the odor source directly, but were exposed to odorized air. In this odor chamber, mice were exposed to linalool odor for 30 min. After the exposure, a behavioral test was given to the mice. For odorless air exposed group, a mouse was placed in the odor chamber as linalool exposed group with only exception that filter papers in the acryl box were not soaked with linalool. To prevent the residual linalool odor, we used another odor chambers for odorless air exposed group. All acryl boxes and cages were changed with respect to each subject and were washed with water and cleaned up with 70% ethanol after daily sessions.

### Light/Dark Box Test

The Light/Dark box apparatus (modified from CPP box, Muromachi Kikai, Tokyo, Japan) was used to measure anxiety (Crawley, 1981). It consisted of an acryl box with two equally sized compartments (25 cm × 18 cm × 21 cm); a light compartment, and a dark compartment. The two compartments were connected with a small entrance (8 cm × 6 cm) and a plastic sheet was spread on the floor of both chambers. The light compartment was illuminated by LED lamp to an intensity of 400 LUX on the floor. The dark compartment was covered with a black lid to shield from light. Mice were placed in the light compartment with their back to the entrance, and behaviors were recorded with a digital video camera for 5 min. The video data was analyzed by EthoVision XT to measure the time spent in light compartment and to measure the number of entries into the light compartment. After each test, the chambers of the light/dark box were washed with water and cleaned up with 70% ethanol.

### Elevated Plus Maze Test

The elevated plus maze apparatus (EPM-04M, Muromachi Kikai, Tokyo, Japan) was used to measure anxiety (Lister, 1987). It consisted of two open arms (30 cm × 6 cm) and two closed arms with walls on the side and the end (30 cm × 6 cm × 15 cm), and central platform (6 cm × 6 cm). The height of the arm was 40 cm from the floor. Illumination was set to 100 LUX on the central platform floor. The mouse was placed on the central platform facing the open arms and was videotaped for 5 min. The video data was analyzed by EthoVision XT to measure the time spent in open arms and the number of entries into open arms and the total distance moved. After each test, the maze was washed with water and cleaned with 70% ethanol.

### Accelerated Rotarod Test

To assess the motor coordination and balance, we performed accelerating rotarod test (Jones and Roberts, 1968) using single lane rotarod apparatus (MK-630B, Muromachi Kikai, Tokyo, Japan). Mice received two trained trials (each trial continued for 300 s with fixed-speed (4 rpm)) with 30 min interval on two consecutive days prior to testing for acclimatization to the apparatus. On the experimental day, after 30 min exposure to odor, mice were placed on the rotating bar and the rotating speed of the rotarod was gradually increased from 4 rpm to 40 rpm within 300 s. The time remained on the rotating bar was measured (Vincenzi et al., 2013).

### Olfactory Epithelium Deprivation

For olfactory deprivation, we disrupted the olfactory epithelium by intraperitoneal administration of 3-MI, which induces extensive destruction of the olfactory mucosa, resulting in anosmia (Kim et al., 2010). Briefly, 300 mg/kg of 3% 3-MI in corn oil was administered by intraperitoneal injection. In control group, 10 mL/kg of corn oil was administered by intraperitoneal injection in control group. Two weeks after the injection, 3-MI treated mice were used for the behavioral test (Tashiro et al., 2016).

### Olfactory Habituation/Dishabituation Test for Anosmia

When an animal smells a novel odor, the animal investigates the odor by approaching and sniffing. With repeated presentations of the odor, the number of approaching to the odor and the time spent for sniffing the odor are progressively reduced (habituates). When the animal is exposed to a novel odor and detects the new odor, the animal shows renewed investigation.

Based on the innate behavior, we performed olfactory habituation/dishabituation tests to confirm whether 3-MI treated mice were anosmic for linalool (Gregg and Thiessen, 1981; Guan et al., 1993; Luo et al., 2002; Woodley and Baum, 2003). A 3-MI treated mouse was placed in a cage (12 cm × 20 cm × 10 cm) with a wire-mesh lid and was exposed to a cotton swab soaked with 20 μL of water three times for 2 min (habituation trials), and then exposed to a cotton swab soaked with 20 μL of linalool for 2 min (dishabituation test trial). Number of approaches and time spent sniffing to the cotton swab were recorded as exploratory behaviors. Approaching was defined as the action of the mouse moving its nose to within 10 mm of the cotton swab. Sniffing was defined as the action of the mouse keeping its nose to within 10 mm of the swab for at least 1 s to smell.

### Data Analyses

If not otherwise specified, statistical comparisons were performed using one-way ANOVA with *post hoc* Tukey’s multiple comparison tests using Prism6 software (GraphPad Software, Inc.). The criterion for statistical significance was *p* < 0.05 in all cases. After one-way ANOVA or unpaired *t*-test, we performed *post hoc* power analyses using G*Power three software (Faul et al., 2007). The raw data supporting the conclusion of this manuscript will be made available by the authors, without undue reservation, to any qualified researcher.

## Results

### Linalool Odor Exposure Induces Anxiolytic Effects in Mice

**Figure 1:**
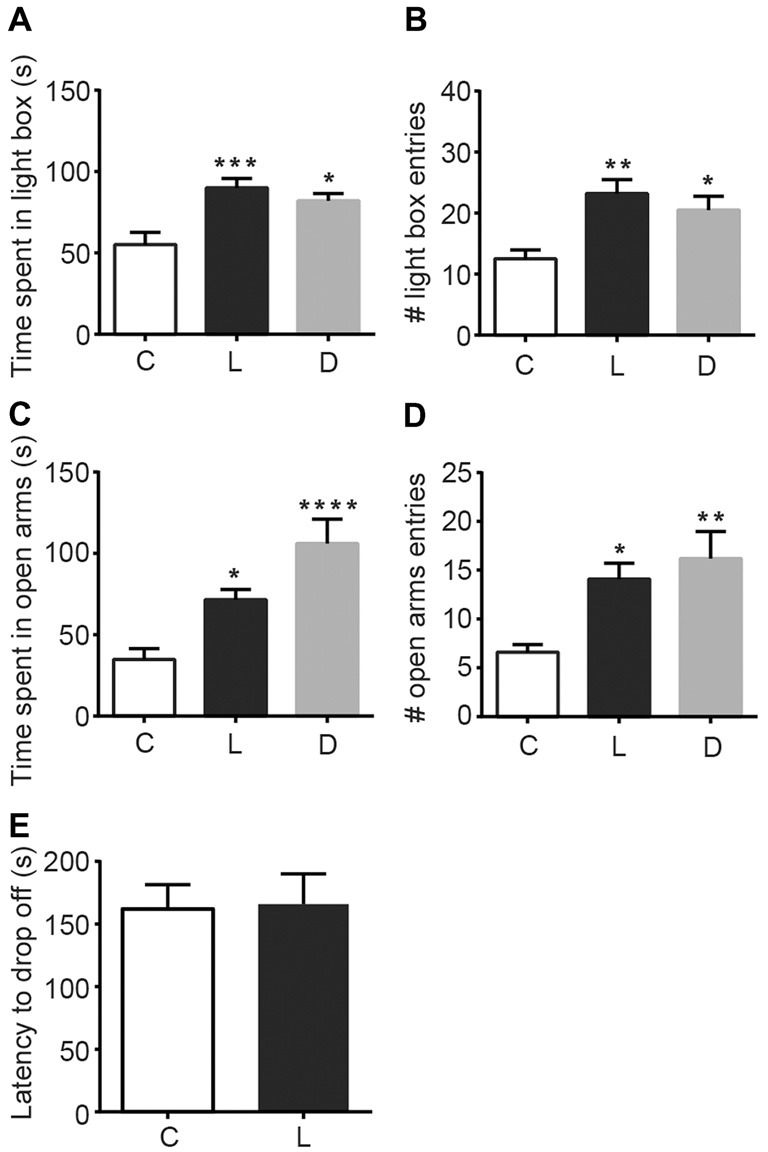
Anxiolytic effects of linalool inhalation and diazepam injection in the Light/Dark box and elevated plus maze (EPM) tests. In the Light/Dark box test, time spent in light box **(A)** and number of entries to light box **(B)** were significantly increased in the linalool group. In the EPM test, time spent in open arms **(C)** and number of entries to open arms **(D)** were significantly increased in the linalool group. *n* = 10 for all groups in **(A–D)**. **(E)** Latency to drop off from accelerating rotarod. *n* = 6 for odorless air group and *n* = 7 for linalool group. **(C)** Mice exposed to odorless air; L, mice exposed to 200 μL of linalool odor. **(D)** Mice administered diazepam intraperitoneally. Each column represents mean ± SEM. *P < 0.05, **P < 0.01, ***P < 0.001, ****P < 0.0001 compared to the odorless air-exposed control group (*post hoc* Tukey’s multiple comparison test).

To examine the anxiolytic effects of linalool odor, we performed classical tests for anxiety immediately after mice were exposed to linalool vapor (Figure 1). Light/Dark box testing revealed that exposure to linalool odor significantly increased exploratory behavior in the light chamber (Figures 1A,B), indicating anxiolytic effects (time spent in light box: *F*_(2,27)_ = 9.184, *p* = 0.0009, *statistical power* = 0.9757 (one-way ANOVA), *p*_control-linalool_ = 0.0010 (Tukey’s multiple comparison test); number of light box entries: *F*_(2,27)_ = 7.317, *p* = 0.0029, *statistical power* = 0.9365 (one-way ANOVA), *p*_control-linalool_ = 0.0029 (Tukey’s multiple comparison test), *n* = 10 per group). The effects were comparable to those induced by diazepam (1.5 mg/kg, i.p.) administration (time spent in light box: *p*_linalool-diazepam_ = 0.6139 (Tukey’s multiple comparison test); number of light box entries: *p*_linalool-diazepam_ = 0.6276). To confirm the anxiolytic effects of linalool odor exposure, we next performed the EPM test (Figures 1C,D). The results showed a significant increase of exploration of the open arms (time spent in open arms: *F*_(2,27)_ = 12.35, *p* = 0.0002, *statistical power* = 0.9958 (one-way ANOVA), *p*_control-linalool_ = 0.0416 (Tukey’s multiple comparison test); number of open arms entries: *F*_(2,27)_ = 6.982, *p* = 0.0036, *statistical power* = 0.9252 (one-way ANOVA), *p*_control-linalool_ = 0.0258 (Tukey’s multiple comparison test), *n* = 10 per group), again indicating anxiolytic effects of linalool odor. Next, to examine whether linalool odor exposure impaired motor function, we performed accelerating rotarod test. The latency to drop off in the linalool odor-exposed group was not significantly different from that in the odorless air-exposed group (*t* = 0.121, *p* = 0.906, *statistical power* = 0.05147 (unpaired *t*-test); *n*_control_ = 6, *n*_linalool_ = 7), suggesting that the coordinated motor skill was not affected by linalool odor exposure (Figure 1E). From these results, we concluded that linalool vapor exposure induced anxiolytic effects without motor impairment in mice.

**Figure 2:**
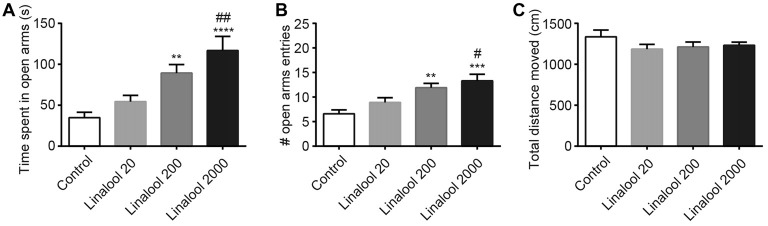
Dose-dependency of anxiolytic effects of linalool odor exposure. Time spent in open arms **(A)** and number of entries to open arms **(B)** indicate that the anxiolytic effects were dependent on the linalool concentration. **(C)** Spontaneous locomotor activity was not impaired by linalool odor exposure. Total distances moved during 5 min in the EPM were not significantly varied. Control, mice exposed to odorless air-exposed mice; Linalool 20, mice exposed to 20 μL of linalool; Linalool 200, mice exposed to 200 μL of linalool; Linalool 2,000, mice exposed to 2,000 μL of linalool; *n* = 10 for all groups. Results are expressed as mean ± SEM. **P < 0.01, ***P < 0.001, ****P < 0.0001 compared to the Control group. ^#^*P* < 0.05, ^##^*P* < 0.01 compared to the Lin 20 group (*post hoc* Tukey’s multiple comparison test).

Next, to examine the dose dependency of the linalool vapor-induced anxiolytic effects, we assessed the effect of several linalool doses using the EPM test. Results indicated that time spent in open arms (Figure 2A) and total number of open arm entries (Figure 2B) increased in accordance with the increase of the linalool dose (time spent in open arms: *F*_(3,36)_ = 10.54, *p* < 0.0001, *statistical power* = 0.9989 (one-way ANOVA), *p*_control-linalool 200_ = 0.008, *p*_control-linalool 2000_ < 0.0001, *p*_linalool 20-linalool 2000_ = 0.0020 (Tukey’s multiple comparison test); number of open arms entries: *F*_(3,36)_ = 8.797, *p* = 0.0002, *statistical power* = 0.9952 (one-way ANOVA), *p*_control-linalool 200_ = 0.0039, *p*_control-linalool 2000_ = 0.0002, *p*_linalool 20-linalool 2000_ = 0.0203 (Tukey’s multiple comparison test); *n* = 10 per group). It should be noted that the total distance moved during the EPM test was not significantly affected by linalool odor exposure (Figure 2C; *F*_(3,36)_ = 1.137, *p* = 0.3472 (one-way ANOVA), *statistical power* = 0.3086; *n* = 10 per group), suggesting that the effects of linalool odor were anxiolytic rather than sedative.

### Linalool Vapor-Induced Anxiolytic Effects Were Triggered by Olfactory Input

**Figure 3:**
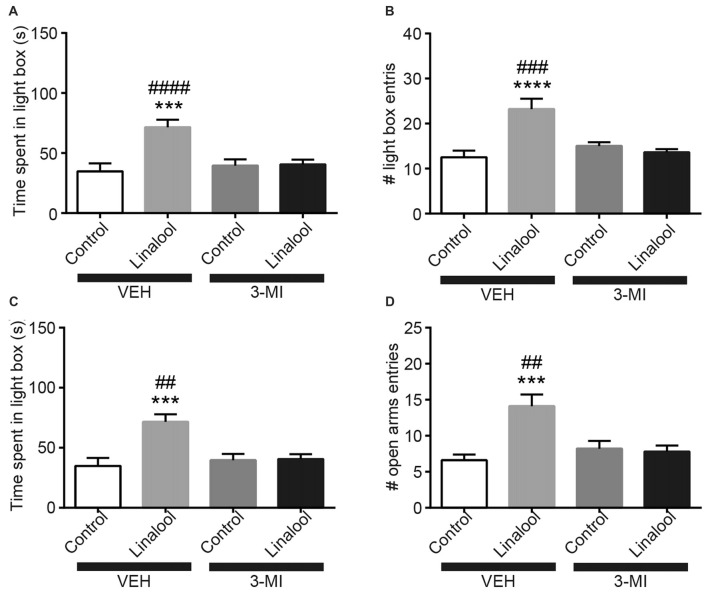
Olfactory input was essential for the anxiolytic effects induced by linalool exposure. The increase in time spent in light box **(A)** and in number of entries to light box **(B)** by linalool odor exposure were not observed in anosmic mice resulting from treatment with 3-methylindole (3-MI). The increase in time spent in open arms **(C)** and number of entries to open arms **(D)** were not observed in the anosmic mice. Control, mice exposed to odorless air; Linalool, mice exposed to 200 μL of linalool; VEH, mice administered vehicle (corn oil, i.p.); 3-MI, mice administered 3-MI (300 mg/kg, i.p.); *n* = 10 for all groups; each column represents mean ± SEM. ***P < 0.001, ****P < 0.0001 compared to the vehicle injected Control group, ^##^*P* < 0.01, ^###^*P* < 0.001, ^####^*P* < 0.0001 compared to the 3-MI injected Linalool group (*post hoc* Tukey’s multiple comparison test).

**Figure 4:**
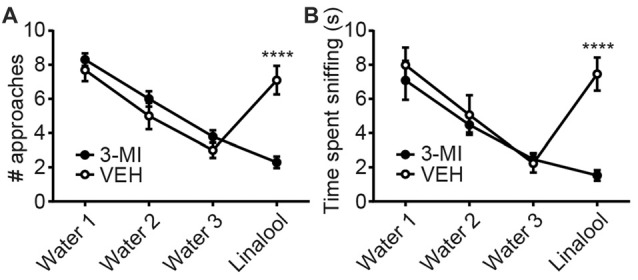
Confirmation of olfactory deprivation caused by 3-MI administration in olfactory habituation/dishabituation test. Number of approaches **(A)** and time spent sniffing **(B)** to the water-soaked cotton swab were not different between vehicle and 3-MI treated mice, but both to the linalool-soaked cotton swab were significantly decreased in 3-MI treated anosmic mice. VEH, mice administered vehicle (corn oil, i.p.); 3-MI, mice administered 3-MI (300 mg/kg, i.p.); Water, distilled water (200 μL); Linalool, Linalool (200 μL). *n* = 10 for all groups. Results are expressed as mean ± SEM. ****P < 0.0001 (*post hoc* Bonferronie’s multiple comparison test).

To examine whether the anxiolytic effects were triggered by olfactory input evoked by linalool odor exposure, we assessed the effects in anosmic mice (Figure 3). In 3-MI administered anosmic mice, the linalool odor-induced anxiolytic effects were not observed in the Light/Dark box test (Figures 3A,B; time spent in light box: *F*_(3,36)_ = 12.75, *p* < 0.0001 (one-way ANOVA), *statistical power* = 0.9998; *p*_(3MI/control-3MI/linalool)_ = 0.9994 (Tukey’s multiple comparison test); number of light box entries: *F*_(3,36)_ = 10.67, *p* < 0.0001 (one-way ANOVA), *statistical power* = 0.9990; *p*_(3MI/control-3MI/linalool)_ = 0.9093 (Tukey’s multiple comparison test)) and the EPM test (Figures 3C,D; time spent in open arms: *F*_(3,36)_ = 8.794, *p* = 0.0002 (one-way ANOVA), *statistical power* = 0.9952; *p*_(3MI/control-3MI/linalool)_ = 0.9994 (Tukey’s multiple comparison test); number of open arms entries: *F*_(3,36)_ = 8.827, *p* = 0.0002 (one-way ANOVA), *statistical power* = 0.9953; *p*_(3MI/control-3MI/linalool)_ = 0.8035 (Tukey’s multiple comparison test); *n* = 10 per group). After the anxiety tests, we assigned 10 3-MI mice and 10 vehicle administered mice to olfactory habituation/dishabituation test. The result showed that exploratory behaviors towards the linalool odor were not observed in 3-MI mice, indicating that 3-MI treated mice could not detect the odor of linalool l (Figures 4A,B; number of approaches to the odor source: *F*_3MI treatment (1,18)_ = 1.132, *p*_3MI treatment_ = 0.3014; *F*_odor (3,54)_ = 36.75, *p*_odor_ < 0.0001; *F*_interaction (3,54)_ = 19.26, *p*_interaction_ < 0.0001 (repeated-measured two-way ANOVA); *p*_linalool/VEH-linalool/3MI_ < 0.0001 (Bonferroni’s multiple comparison test); time spent sniffing of the odor source: *F*_3MI treatment (1,18)_ = 5.400, *p*_3MI treatment_ = 0.0320; *F*_odor (3,54)_ = 18.09, *p*_odor_ < 0.0001; *F*_interaction (3,54)_ = 7.817, *p*_interaction_ = 0.0002 (repeated-measured two-way ANOVA), *p*_linalool/VEH-linalool/3MI_ < 0.0001 (Bonferroni’s multiple comparison test), *n* = 10 per group).

### The GABAergic System Mediates the Linalool Odor-Induced Anxiolytic Effects

**Figure 5:**
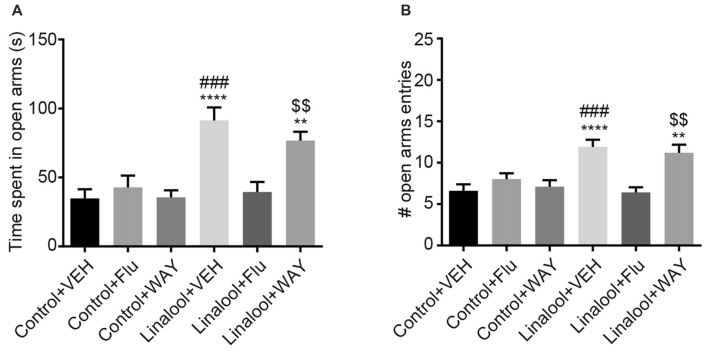
Influence of pretreatment with flumazenil or WAY100635 on the anxiolytic effects of linalool. The increase in time spent in open arms **(A)** and number of entries to open arms **(B)** from linalool odor exposure were eliminated in mice treated with flumazenil, but not with WAY100635 in linalool odor-induced anxiolytic effects. Pretreatment with flumazenil or WAY100635 without linalool odor exposure did not affect exploratory behaviors. Control, mice exposed to odorless air; Linalool, mice exposed to 200 μL of linalool; VEH; mice administered vehicle (2% Tween80 in saline); Flu, mice administered flumazenil (3 mg/kg i.p.); WAY, mice administered WAY100635 (0.5 mg/kg i.p.); each column represents mean ± SEM; *n* = 10 for all groups. **P < 0.01, ****P < 0.0001 compared to the Control + VEH group, ^###^*P* < 0.001 compared to the Linalool + Flu, ^$$^*P* < 0.01 compared to the Control + WAY (*post hoc* Tukey’s multiple comparison test).

The beneficial effects of BDZs and azapirones in treating anxiety disorders indicate the involvement of BDZ-responsive GABA_A_Rs and 5-HT_1A_Rs. To assess whether those receptors were involved in the linalool odor-induced anxiolytic effects, we performed the EPM test to examine the linalool odor-induced anxiolytic effects, but administered either flumazenil (antagonist for BDZ site of GABA_A_Rs) or WAY100635 (5-HT_1A_R antagonist) before the test (Figure 5). Pretreatment of flumazenil completely abolished the anxiolytic effects of linalool odor, indicating that GABAergic transmission via BDZ-responsive GABA_A_Rs was essential for the anxiolytic effects (time spent in open arms: *F*_(5, 54)_ = 10.70, *p* < 0.0001 (one-way ANOVA), *statistical power* = 0.9999, *p*_linalool/VEH-linalool/Flu_ = 0.0001, *p*_control/Flu-linalool/Flu_ = 0.9995 (Tukey’s multiple comparison test); number of open arms entries: *F*_(5,54)_ = 8.966, *p* < 0.0001 (one-way ANOVA), *statistical power* = 0.9999, *p*_linalool/VEH-linalool/Flu_ = 0.0002, *p*_control/Flu-linalool/Flu_ = 0.7239 (Tukey’s multiple comparison test); *n* = 10 per group). In contrast, WAY100635 treatment induced no significant changes to the anxiolytic effects of linalool odor (time spent in open arms: *p*_linalool/VEH-linalool/WAY_ = 0.7374, *p*_control/WAY-linalool/WAY_ = 0.0032 (Tukey’s multiple comparison test); number of open arms entries: *p*_linalool/VEH-linalool/WAY_ = 0.9895, *p*_control/WAY-linalool/WAY_ = 0.0086 (Tukey’s multiple comparison test)), suggesting that serotonergic transmission via 5-HT_1A_R may not be involved in the effects.

## Discussion

### Technical Limitations of This Study

In this study, we showed the linalool odor-induced anxiolytic effects only in adult male mice. To have a general understanding of the phenomena, we further need to assess the effects on female (for examining the sex differences) and on younger/elder mice (for examining the age dependency).

### Olfactory Input Evoked by Linalool Odor Induced Anxiolytic Effects

In this study, we found that exposure to linalool odor induced anxiolytic effects in mice (Figure 1). The effects were not observed in anosmic mice (Figure 4), indicating that the effects were triggered by olfactory input evoked by linalool odor exposure. Previously, several studies have examined that linalool inhalation induced anxiolytic effects (Linck et al., 2010; Takahashi et al., 2011; Zhang et al., 2016). However, because the contribution of the olfactory system was not directly examined, the nature of how linalool may induce the effects was not revealed. In this study, we confirmed that the olfactory system was essential for the linalool odor-induced anxiolytic effects using anosmic mice (Figures 3, 4). Thus, we established that the olfactory input triggered by linalool odor was responsible for inducing the anxiolytic effects.

In addition to linalool, several other odors have also been reported to induce anxiolytic effects when inhaled. For example, inhalation of (+)-limonene Lima et al. (2013), linalool oxide Souto-Maior et al. (2011), or α-pinene (Satou et al., 2014) were shown to reduce anxiety in mice. In these studies, authors did not address the contribution of olfactory input to the anxiolytic effects. However, inhalation of these odorous compounds may trigger the anxiolytic effects via olfactory input. It is noteworthy that the effects resulting from limonene inhalation were not antagonized by pre-treatment of flumazenil (Lima et al., 2013). Taken together with our results, it is possible that there may be at least two parallel anxiolytic pathways involving BDZ-responsive GABA_A_Rs-dependent, and -independent systems evoked by olfactory input.

Due to the fact that a given odorant receptor is activated by a range of odor molecules with similar structure(s) (Malnic et al., 1999), a range of odor molecules may also trigger specific central neuronal circuits required for linalool-induced anxiolytic effects. For further analyses, identification of the odorant receptor(s) contributing to the odor-induced anxiolytic effects and a systematic survey of odorants which act upon the receptor(s) would be beneficial. In addition to the odorant receptors, T-type calcium channels (TTCCs) are also affected by linalool (El Alaoui et al., 2017). Because the TTCCs contribute to the generation of action potentials in olfactory sensory neurons (Kawai et al., 1996), the modulation of TTCCs by linalool may also contribute to linalool odor-induced analgesia.

Several studies have previously reported that systemic administration of linalool intraperitoneally (Umezu et al., 2006; Coelho et al., 2011; Guzman-Gutierrez et al., 2012) or orally (Cheng et al., 2015) induced anxiolytic effects. In these studies, the primary sites that are affected by linalool were not addressed. However, it has been assumed that linalool entering the bloodstream via absorption through the airway may modulate the glutamatergic neurotransmission (Elisabetsky et al., 1995; Batista et al., 2008). Another possibility raised by our results is that systemically administrated-linalool may be emitted in the exhaled breath and drive the anxiolytic effects via olfactory system retronasally (Kikuta et al., 2016).

### Neuronal Circuits Underlying the Linalool Odor-Induced Anxiolytic Effects

In this study, we showed that flumazenil administration completely abolished the linalool odor-induced anxiolytic effects (Figure 5). Flumazenil blocks the GABA-induced anxiolytic effects by antagonizing the BDZ site of α2-GABA_A_R (Rudolph et al., 1999; Low et al., 2000). Because there were no longer anxiolytic effects from linalool odor when olfactory deprived mice were used (Figure 3), the effects may not be evoked by direct activation of BDZ sites with linalool, but rather by activation of intrinsic anxiolytic circuits involving GABAergic transmission via BDZ-responsive GABA_A_R.

In contrast to the antagonism of flumazenil, 5-HT_1A_R antagonist WAY100635 did not show any significant changes to linalool odor-induced anxiolytic effects (Figure 5B). Clinical and preclinical studies have indicated that the serotonergic system which includes 5-HT_1A_R, also plays a key role in modulating anxiety and is one of the major targets of the clinical treatment targets (Gordon and Hen, 2004; Albert et al., 2014). On the other hand, our findings suggest that the serotonergic transmission via 5-HT_1A_R may not be involved in the anxiolytic effects induced by linalool odor.

In summary, we found that linalool odor exposure induced anxiolytic effects without motor impairment in mice. The effects were abolished in anosmic mice, indicating that olfactory input evoked by linalool odor was necessary to trigger the effects. Furthermore, synaptic transmission with BDZ-responsive GABA_A_Rs was also essential for the effects. These findings give us a foundation towards clinical application of linalool odor for anxiety disorders. Moreover, linalool odor-induced anxiolytic effects may be applicable for preoperative patients because pretreatment with anxiolytics can alleviate preoperative stress and thus contribute to place patients under general anesthesia more smoothly. In addition, for patients who may have difficulties with oral or suppository administration of anxiolytics, such as infants, utilizing linalool odor to help reduce anxiety may be a convenient and promising alternative.

## Author Contributions

HH, TK and HK designed the study. HH and HK conducted the study and analyzed the data. HH, YK, TK and HK wrote the article. All authors reviewed the manuscript.

## Conflict of Interest Statement

The authors declare that the research was conducted in the absence of any commercial or financial relationships that could be construed as a potential conflict of interest.
